# Genetic screening in cohort of Egyptian patients with pulmonary arterial hypertension disease

**DOI:** 10.1007/s11845-025-03889-5

**Published:** 2025-02-11

**Authors:** Samar I. E. Ayyad, Miral M. Refeat, Engy A. Ashaat, Abdel-Rahman B. Abdel-Ghaffar, Germine M. Hamdy

**Affiliations:** 1 Helwan Joint Laboratory, Helwan, Egypt; 2 Medical Molecular Genetics Department, Human Genetics and Genome Research Institute, National Research Centre, Cairo, 12622 Egypt; 3 Clinical Genetics Department, Human Genetics and Genome Research Institute, National Research Centre, Cairo, 12622 Egypt; 4 Biochemistry Department, Faculty of Science, Ain Shams University, Cairo, Egypt

**Keywords:** *BMPR2* gene, Pulmonary arterial hypertension disease, Sanger sequencing, Variants

## Abstract

**Background:**

Variants in the bone morphogenetic protein 2 receptor gene (*BMPR2*) are the most frequent genetic cause of pulmonary arterial hypertension (PAH). However, correlation of *BMPR2* variants and PAH clinical phenotype remains to be elucidated.

**Methods and results:**

The goal of the present study is to investigate variants of the causative gene (*BMPR2*) in 25 Egyptian patients clinically pre-diagnosed with PAH symptoms and 10 healthy candidates using Sanger sequencing technique. Three pathogenic heterozygous missense variants have been illustrated in *BMPR2* gene, two novel variants (V387E, E481K) in exon 9 and 11 respectively and one previously reported missense variant (C496G) in exon 11. The remaining 22 patients as well as the 10 healthy individuals showed no pathogenic variants.

**Conclusion:**

Further studies on larger number of participants, using advanced NGS technique, should be performed to enrich information about genotype/phenotype correlations and incidence of PAH disease among Egyptian population; thus, it would provide families of PAH patients with accurate genetic counseling in order to prevent disease recurrence.

## Introduction

Pulmonary hypertension (PH) is a progressive, chronic, and challenging disease that influences about 1% of the global population and it is considered to be life-threatening condition that characterized by abnormal elevated blood pressure of the pulmonary circulation, which results, over time, into disability, and even death [1]. PH is classified according to clinical presentation, pathological findings, and hemodynamic characteristics into five groups; pulmonary arterial hypertension (PAH), which embraces various diseases including idiopathic, heritable, persistent PH of the newborn syndrome, in addition to PAH associated diseases such as connective tissue disease, drug- and toxin-induced PAH disease, congenital heart diseases, portal hypertension, human immunodeficiency virus (HIV), hemoglobinopathies, and schistosomiasis. The other 4 groups of PH are due to left heart disease (LHD); lung disease; chronic thromboembolic PH (CTEPH); and PH due to multifactorial mechanisms [2]. Pulmonary arterial hypertension (PAH) is a rare, devastating subtype condition of PH with an incidence of 15 to 60 per million, affecting the small pulmonary arteries, and though increases resistance to blood flow through the pulmonary vasculature leading to right ventricular failure, and death [3]. Prevalence of PAH represent 15 cases/100,000 Australians, 0.37 cases/100,000 French citizens, 1.4 cases/100,000 American cases, and 0.008/100,000 Finch individuals [4]. Variants in the bone morphogenetic protein type 2 receptor genes (*BMPR2*) are the main cause of PAH disease. These variants have been recognized in 11 to 40% sporadic cases of idiopathic PAH and about 53.3–80% within heritable pulmonary arterial hypertension (HPAH) patients [5]. Indeed, PAH patients with *BMPR2* mutations show higher incidence than those resulted from mutations in other genes such as *KCNK3*, *ALK1*, and *CAV1*. *BMPR2* is a 190-kb gene comprising 13 exons that encode bone morphogenetic proteins (BMPs). These proteins play an essential role through BMP receptors (BMPRs) during embryonic development and adult homeostasis. Recently, more than 600 mutations have been described in *BMPR2* gene [6]. Clinical criteria of PAH patients are complex and involve endothelial dysfunction, chronic inflammation, smooth muscle cell proliferation, pulmonary arteriole occlusion, antiapoptosis, and pulmonary vascular remodeling. Patients suspected to PAH should be referred to an expert PH clinician to manage the disease and its comorbidities (e.g., sleep apnea and COPD) [7]. Additionally, PAH patients require supportive therapies such as diuretics, oxygen, and management of HF, including treatment of aggravating factors, optimization of fluid status, reduction of RV after load, and cardiac inotropes if indicated. Iron deficiency is common in PAH, and monitoring of iron levels, with iron substitution, when necessary, is indicated [8]. A combination therapy of endothelin receptor antagonist, a phosphodiesterase type 5 (PDE5) inhibitor, and a prostaglandin I2 (PGI2) analog was able to improve the prognosis of certain patients with idiopathic PAH. Low-level rated exercise, as walking and oxygen supplementation to save saturation over 90% at rest and with effort, sleep, or altitude are advisable [9]. In the current study, 25 Egyptian patients clinically diagnosed with pulmonary arterial hypertension (PAH) and 10 healthy candidates have been screened for pathogenic variants in *BMPR2* genes using direct Sanger sequencing.

## Subjects and methods

### Subjects

The present study enrolled 25 unrelated patients (nine females 36% and 16 males 64%) of age ranged from 3 days to 5 years, and ten healthy candidates their age ranged from few days up to 6 years old. Patients were referred from Clinical Genetics Clinic at the Medical Research of Excellence Centre, National Research Centre (NRC), Cairo, Egypt. Most of them were offspring of consanguineous marriages. The parents signed an informed consent approved by the Medical Research Ethics Committee of NRC.

#### Clinical criteria

All patients were subjected to family history, drug investigations, laboratory tests, clinical assessment, radiological examination as chest radiograph, echocardiography, and ECG. They were clinically tested according to the guidelines of PAH diagnosis (up to 25 mmHg) in the WHO/European Respiratory Society/European Society of Cardiology and investigations were confirmed with right heart catheterization (RHC). Patients who refused the procedure of RHC were excluded.

### Molecular analysis of *BMPR2* gene

#### Genomic DNA extraction

Genomic DNA was extracted from peripheral blood of all 25 patients as well as the 10 healthy individuals using Thermo Scientific Gene JET Genomic DNA Purification Kit (Thermo Scientific, USA) according to the manufacturer’s instructions. Concentration and purity of genomic DNA was quantified using Nano drop and stored in aliquots at − 20 °C.

#### PCR amplification

Targeted fragments of identified variants in *BMPR2* gene were amplified using Qiagen Taq PCR Core kit according to manufacturer’s instructions and specific primers that were designed primer3 software (http://bioinfo.ut.ee/primer3-0.4.0/), referring to genomic sequence (GenBank accession numbers). The sequences of the primers are accessible on request. PCR cycling conditions were generated in Perkin-Elmer as follows: 94 °C for 10 min followed by 35 cycles of 94 °C for 1 min, 59 °C for 1 min, and 72 °C for 1 min, finally cycles are extended at 72 °C for 10 min, and then stored at 4 °C.

#### Purification of PCR products

Amplicons were purified using enzymatic treatment Exonuclease/Shrimp Alkaline phosphatase (Sigma, USA) that were held in cycler (Perkin-Elmer) for one cycle at 37 °C for 60 min and 80 °C for 15 min.

#### Sanger sequencing

Purified products were sequenced in both directions using big-dye terminator kit (Applied Biosystems, Foster City, CA, USA) and analyzed on system (ABI 3130 Genetic Analyzer, Applied Biosystems, USA).

#### In silico bioinformatics tools

The achieved sequences were analyzed to identify the pathogenicity and the effect of the variants by first comparing them with genomic reference sequence, using FinchTV 1.4.0 software, then using MetaLR logistic regression (LR), REVEL ensemble method as well as AlphaMissense and BayesDel software to predict the pathogenicity of the missense variants. To investigate the maximum frequency of the variants, dSNP and 1000 genomes populations public databases (dpSNAP and 1000 genome project) were used. EVE software is a deep learning model trained on amino acid sequences for the prediction of clinical significance of human variants; meanwhile, PolyPhen2 (Polymorphism Phenotyping V2) and SIFT (Sorting Intolerant from Tolerant) analysis software predict the effect of amino acid substitution on protein function. Mutation Taster (http://MutationTaster.org) was used to evaluate DNA sequence variants for their disease-causing potential.

## Results

### Clinical investigations

**Fig. 1:**
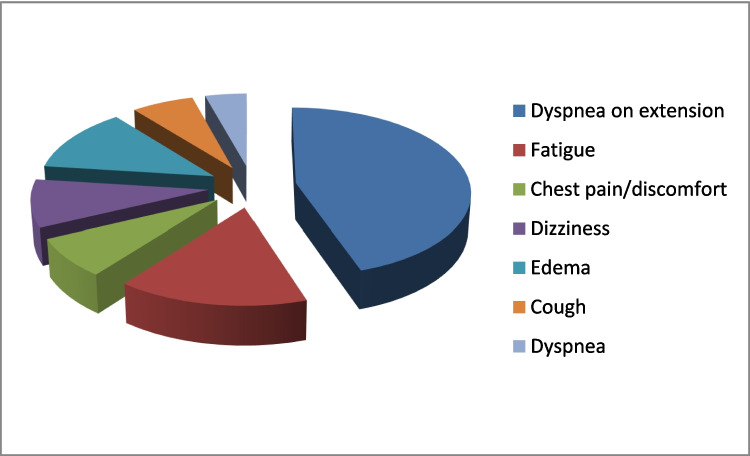
Clinical symptoms of PAH patients

Clinical criteria of included patients revealed dyspnea on extension (84%), fatigue (29%), chest pain/discomfort (14%), dizziness (17%), fainting spells, edema (23%), cough (12%), bluish skin, and angina. Pediatric cases of age from the neonatal period to adulthood suffered prenatal etiological factors, as well as postnatal parenchymal and vascular abnormalities in lung development (Fig. 1).

### Molecular analysis

**Fig. 2:**
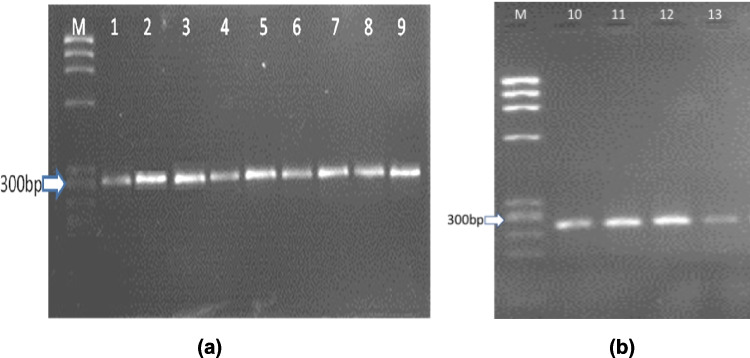
2% agarose gel electrophoresis of the 13 amplified fragments of *BMPR2* gene. **a** Fragments 1–9. **b** Fragments 10–13. M: DNA marker

PCR amplification was performed for the targeted variants of *BMPR2* gene in all included subjects, 25 patients and 10 candidates. A representative example of the amplified fragments of *BMPR2* gene for one patient is shown on a 2% agarose gel (Fig. 2).

**Fig. 3:**
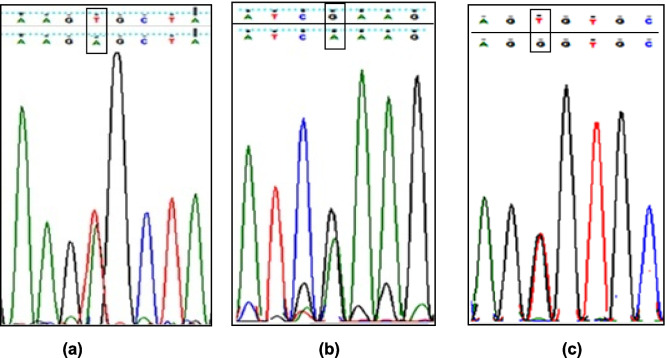
Electropherograms of the 3 pathogenic mutations described in *BMPR2* gene. **a** Novel missense mutation (V387E) identified in exon 9 of patient 1. **b** Novel missense mutation (E481K) detected in exon 11 of patient 2. **c** Missense mutation (C496G) detected in exon 11 of patient 3

Sanger sequencing revealed 3 different heterozygous pathogenic missense variants in 3 unrelated patients. The analysis of the three detected variants using public databases (dpSNAP and 1000 genome project), PolyPhen2 (Polymorphism Phenotyping V2), and SIFT (Sorting Intolerant from Tolerant) software revealed 2 novel mutations in exon 9 and 11, while the third one was reported in exon 11 previously. The remaining 22 patients, as well as the 10 healthy candidates, showed no pathogenic variants in the analyzed gene. The first male case possessed a novel heterozygous missense variant [c.1160 T > A; V387E] in exon 9 resulted in valine to glutamic acid residue substitution. The other two male patients carried 2 heterozygous missense variant in exon 11, the novel one [1441G > A; E481K], led to amino acid substitution of glutamic acid with lysine residue. Meanwhile, the other missense variant was previously reported [c.1486 T > G; C496G] and caused cysteine to glycine residue substitution (Fig. 3, Table 1).

**Table 1:** Clinical and molecular investigations in the three unrelated patient

Patient	Gender	Age	Consanguinity	Family history	Clinical criteria	Anthropometric	PH	DNA changes	A.A	Exon	Mutation type
1	Male	3 years	-Ve	Siblings died	Fatigue, chest pain, edema, hard spelling	On mean	+ Ve	c.1160 T > A	V387E	9	Missense
2	Male	4 years	-Ve	Siblings died	ASD, PH, dizzy, swelling in legs and ankles, fast heart pulses, and tiredness	On mean	+ Ve	c.1441G > A	E481K	11	Missense
3	Male	3 days	-Ve	Siblings died	PDA, PAH, shortness of breath, palpitation, angina, high mean of pulmonary artery pressure	On mean	+ Ve	c.1486 T > G	C496G	11	Missense
4–25	13 males and 9 females	Few days up to 6 years	-Ve	Siblings died	Mean of pulmonary artery pressure > 3.3 kPa (25 mmHg)	On mean	+ Ve	No mutation detected			

*CHD*, congenital heart disease; *ASD*, atrial septal defect; *PDA*, patent ductus arteriosus

### Functional analysis of variants using software tools

Mutation Taster evaluated all variants as disease-causing potential; however, pathogenicity of missense variants varied as deleterious (low, moderate, and strong) using REVEL, AlphaMissense, and BayesDel softwares. According to REVEL method and BayesDel software, the 3 variants were moderate score in the range 0.86 to 0.89 and 0.3 to 0.5 respectively; meanwhile, AlphaMissense showed strong pathogenicity (> 0.9840). Scores of EVE software ranged from 0.7 to 0.9 which reflected the deleterious pathogenicity. PolyPhen2 and SIFT analysis software predicted that the 3 variants were of probably damaging effect on the protein function. Meta (LR) score ranged from 0.4 to 0.8, as higher values are more likely of being deleterious (Table 2).

**Table 2:** Bioinformatics analysis of the 3 enclosed mutations

Patient	Variant	ACMG classification	dbSNP	Aggregated prediction	REVEL	EVE	AlphaMissense	SIFT	Polyphen2	MetaLR	BayesDel	Mutation Taster
1	c.1160 T > A V387E	PM2, PM1, PP3	NA	Deleterious (0.87)	Deleterious (moderate) (0.89)	Deleterious (0.97)	Deleterious (strong) (0.997)	Deleterious (supporting) (0)	Probably damaging Sensitivity: 0.14 Specificity: 0.99	Deleterious (low) (0.61)	Deleterious (moderate) (0.33)	Disease causing
2	c.1441G > A E481K	PM2, PM1	rs767730208	Uncertain (0.6)	Deleterious (moderate) (0.86)	Deleterious (0.79)	Deleterious (strong) (0.937)	Deleterious (supporting) (0)	Probably damaging Sensitivity: 0.00 Specificity: 1	Deleterious (low) (0.4)	Deleterious (moderate) (0.58)	Disease causing
3	c.1486 T > G C496G	PM2, PM5, PM1, PP3	N/A	Deleterious (0.86)	Deleterious (moderate) (0.87)	Deleterious (moderate) (0.85)	Deleterious (strong) (0.984)	Deleterious (supporting) (0)	Probably damaging Sensitivity: 0.00 Specificity: 1	Deleterious (moderate) (0.84)	Deleterious (moderate) (0.49)	Disease causing

*HGVS*, Human Genome Variation Society; *N/A*, not applicable; *PVS*, pathogenic very strong; *PS*, pathogenic strong; *PM*, pathogenic moderate; *PP*, pathogenic supporting

## Discussion

Pulmonary hypertension (PH) is a pathophysiological disorder that could be associated with different cardiovascular and respiratory diseases [10]. Pulmonary arterial hypertension (PAH) is a rare type of PH that affects the pulmonary vasculature with 26–100/million adults worldwide [11]. PAH is the most common PH disease in Egypt; PAH patients with connective tissue diseases (CTDH) represent 16% among PH groups [12]. The current study enrolled 25 patients (nine females and 16 males) of age ranged from 3 days to 5 years, pre-diagnosed with symptoms consistent with clinical criteria of PAH [1]. Patients with *BMPR2* variants present at a younger age are at high risk of death, compared with other gene variants [3]. PAH symptoms include exertional dyspnea, shortness of breath, dizziness, fainting spells, rapid heartbeats, bluish skin, fatigue, weakness, angina, presyncope, and syncope, fluid retention that results in abdominal distention and ankle edema [13]. PAH was estimated with a mean pulmonary artery pressure of 25 mm Hg or more and its pathogenesis is intricate chronic inflammation, pulmonary arteriole occlusion, smooth muscle cell proliferation, and pulmonary vascular remodeling [14]. Pediatric PAH could be presented at age ranged from the neonatal period to adulthood. It is characterized with some unique features that are not found in adults, including prenatal etiological factors, postnatal parenchymal and vascular abnormalities in lung development [15]. Diagnosis of PAH is mostly delayed due to the overlapping of warning signs with other diseases [16]. In the current study, 3 different heterozygous pathogenic missense variants in 3 unrelated patients, 2 novel variants in exon 9 and 11 and one previously reported in exon 11, were analyzed using different bioinformatic tools. Twenty-two patients as well as 10 healthy individuals showed no pathogenic variants in the analyzed gene. This gene encodes a cell membrane type II receptor of the transforming growth factor-signaling pathway, which regulates expression of many target genes [17]. The first male patient included in the current study possessed a novel heterozygous missense variant [c.1160 T > A; V387E] in exon 9 resulted in valine to glutamic acid residue substitution; this variant could affect DNA-transcription process resulting in altering protein features and though its expression, which in turn disrupt the function of the corresponding protein and cause [PAH] diseases. The other two male patients comprised 2 heterozygous missense variants in exon 11, one was novel variant [1441G > A; E481K] and led to amino acid substitution of glutamic acid with lysine residue; meanwhile, the other missense variant was previously reported [c.1486 T > G; C496G] and caused cysteine to glycine residue substitution. The transforming growth factor-β (TGF-β) super family comprises two main branches, TGF-β–activin–nodal branch and bone morphogenetic protein (BMP)–growth differentiation factor (GDF) branch. Substitution variants (deleted or inserted tandem repeats) in *BMPR2* gene lead to reduction in the gene expression functional assessment and dysfunction in the balance between the two branches, yet, resulting in disease progression [18]. According to polyphene-2 program and SIFT software, the 2 novel variants were predicted to degrade protein domain; this resulted in deficiency of gene expression activity with a score of 0.999 (sensitivity: 0.14; specificity: 0.99) [18]. Nevertheless, C496G missense variant was initially reported by Machado et al. in exon 11 of *BMPR2* gene [19] that caused cysteine to glycine residue substitution. Cysteine substitutions comprise most missense variants in the extracellular ligand-binding domain and are concentrated on nine of 10 conserved residues, which are essential for the formation of five disulfide bridges necessary to maintain the integrity of this highly ordered three-dimensional structure. These substitutions might manipulate the side chain packing at the surface of the kinase domain. The occurrence of apparently benign variants at the protein surface suggests a potential role for some of these residues in protein–protein interactions [20]. Asian study identified that the highest incidence of PAH variants was found in the *BMPR2*, *ATP13A3*, and *GDF2* genes using whole exome sequencing (WES) in Asian idiopathic and heritable in a total of 69 patients [21]. According to the Registry to Evaluate Early and Long-Term PAH Disease Management (REVEAL registry), 21% of PAH patients within 2 years are usually diagnosed in late course of the disease, due to its rarity and diversity of clinical presentation. As more clinical data becomes offered, progression of understanding the development of PAH disease will continue. Furthermore, diagnostic algorithm should be used to facilitate efficient diagnostic process [22]. Multiple mechanisms occur at the cellular and tissue level during the disease course, these physiological alterations result in excessive vasoconstriction, medial hypertrophy, intimal fibrosis, and formation of plexiform lesions. Contemporary PAH therapies target one of three major pathways implicated in disease progression: nitric oxide (NO), endothelin-1 (ET-1), and prostacyclin (PGI2) pathways. PAH prognosis is often poor, lack of drugs that can effectively prevent the progression in treatment of PAH. Therefore, it is of great value to find biomarkers that can reflect the condition and prognosis [23]. A notable study conducted by Department of Chest Diseases, Air Forces Specialized Hospital of the Medical Academy on 150 patients with PH, including the 5 groups, revealed that the most frequent PH etiologies were PAH and chronic thromboembolic PH. PAH patients received targeted therapy and demonstrated considerable improvement in decreasing disease severity (WHO Function Class). However, lack of the follow-up and survival data of patients led to limitation in these studies [24].

## Conclusion

In conclusion, variant detection rate in this study was 0.15% (3/25 families). This study presents an initial step to highlight the prevalence of PAH disease in Egypt. The finite results of this study were due to the lack of awareness of patients with the disease and geographical complexity in some rural areas or small cities, led to discontinuous follow-up of clinician and geneticist with patients as well as the need for further advanced next generation techniques as whole exome sequencing to be able to identify wide range of coding and non-coding variants. This will provide affected families with precise diagnosis, which is essential for genetic counseling and for the delivery of the best health care, support, and eventually treatment.

## Data Availability

The data that supports the findings in this study are available from the corresponding author upon reasonable request.
